# Determination of Fetal Left Ventricular Volume Based on Two-Dimensional Echocardiography

**DOI:** 10.1155/2017/4797315

**Published:** 2017-10-23

**Authors:** Li Yu, Yi Guo, Yuanyuan Wang, Jinhua Yu, Ping Chen

**Affiliations:** ^1^Department of Electronic Engineering, Fudan University, Shanghai 200433, China; ^2^Key Laboratory of Medical Imaging Computing and Computer Assisted Intervention of Shanghai, Shanghai 200433, China; ^3^Department of Ultrasound, The First Maternal and Infant Health Care Hospital, Shanghai 200433, China

## Abstract

Determination of fetal left ventricular (LV) volume in two-dimensional echocardiography (2DE) is significantly important for quantitative analysis of fetal cardiac function. A backpropagation (BP) neural network method is proposed to predict LV volume more accurately and effectively. The 2DE LV border and volume are considered as the input and output of BP neural network correspondingly. To unify and simplify the input of the BP neural network, 16 distances calculated from the border to its center with equal angle are used instead of the border. Fifty cases (forty frames for each) were used for this study. Half of them selected randomly are used for training, and the others are used for testing. To illustrate the performance of BP neural network, area-length method, Simpson's method, and multivariate nonlinear regression equation method were compared by comparisons with the volume references in concordance correlation coefficient (CCC), intraclass correlation coefficient (ICC), and Bland-Altman plots. The ICC and CCC for BP neural network with the volume references were the highest. For Bland-Altman plots, the BP neural network also shows the highest agreement and reliability with volume references. With the accurate LV volume, LV function parameters (stroke volume (SV) and ejection fraction (EF)) are calculated accurately.

## 1. Introduction

Two-dimensional echocardiography (2DE) is the most commonly used technique for evaluation of fetal cardiac function due to its nonradiation, low-cost, and quick acquisition. Accurate quantification of left ventricular (LV) volume is of significant importance for fetal cardiac function analysis [1–3]. At present, several methods based on single plane or biplane have been proposed to determine LV volume in 2DE [4]. Generally, the single plane refers to 4-chamber view, and the biplane combines 4-chamber view and 2-chamber view [5]. In clinical examination, a single plane is easier to obtain. Hence, single plane-based volume calculation methods are common. The single plane-based methods rely on adequate LV border definition and geometric assumptions to convert 2D area into 3D LV volumes. The fetal LV border can be extracted by manual delineation or automatic segmentation algorithm [6, 7].

**Figure 1:**
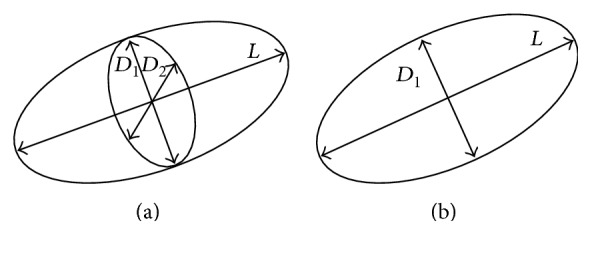
Geometric assumptions for LV in the area-length method. (a) The geometric assumption for biplane area-length method. (b) The geometric assumption for single plane area-length method. *L* is the long-axis, and *D*_1_ and *D*_2_ are the minor axes taken from 4-chamber view and 2-chamber view.

The most classical methods are the area-length method and Simpson's method [8–13]. The area-length method [6, 8] assumes that the left ventricle is an ellipse, as shown in Figure 1(a). *L* is the long axis. *D*_1_ and *D*_2_ are the minor axes taken from perpendicular projections. The long-axis length is measured between the furthest point of the apes to the midpoint of the straight line crossing the mitral valve annulus. For a single plane area-length method, LV is considered as a perfect ellipse, as shown in Figure 1(b), and *D*_1_ and *D*_2_ are equal. The assumption that the LV is a completed ellipse is too idealistic to be true of the actual LV geometry.

**Figure 2:**
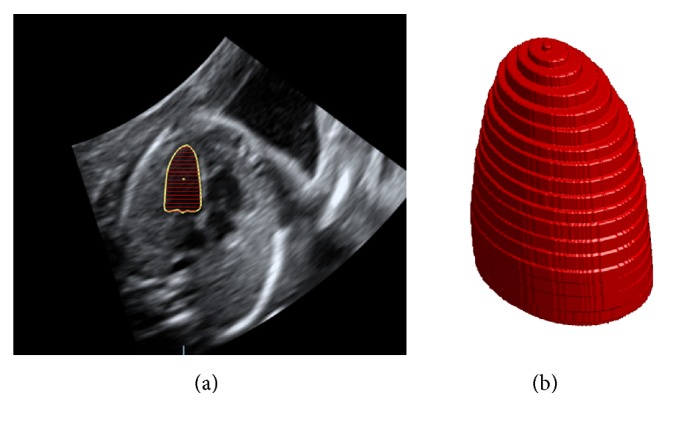
Single plane Simpson's method. (a) Yellow solid contour is the border of LV. Red lines are axes for different individual cylinders. (b) 3D LV model by Simpson's method.

To assume the LV geometry closer to the real geometry, the Simpson's method [9–13] considers the LV as a stack of individual short cylinders. As shown in Figure 2, the LV volume is calculated as the summation of the volumes of each individual cylinder (from the furthest point of the apes to the mitral valve). The LV geometric assumption for Simpson's method is closer to the real geometry than that of the area-length method.

Although the area-length method and Simpson's method are widely used clinically, both of them are based on pure geometric assumptions and mathematical formulas. However, the structure of each fetal LV is complex and variable. Hence, these two models are different from the actual geometry and hard to cover all types of cases.

The determination of fetal LV volume is based on 2DE four-chamber view, which predicts the 3D LV volume from 2D LV border. So, the determination of LV can be regarded as a prediction problem which maps nonlinear relations between the input (2D LV border) and the output (3D LV volume). Recently, a lot of interesting work has been done in the application of machine learning (ML) algorithms for prediction problems. The backpropagation (BP) neural network [14, 15] which has strong nonlinear mapping ability is one of the most effective and accurate methods for prediction.

In this paper, a novel LV volume determination method based on a BP neural network is proposed to determine the LV volume. The complex mapping relations between 2DE LV border and 3D LV volume are learned and stored in the BP neural network. To simplify and unify the input of BP neural network, 16 distances extracted from LV border are used as the inputs. The volume references calculated from the VOCAL II mode in 4D view are used as the labels for BP neural network training. To illustrate the advantages of the BP neural network for calculation of LV volume, several conventional methods were compared.

## 2. Materials and Methods

### 2.1. Participants

The study population comprises 50 pregnant women between 20 and 28 gestational weeks, who underwent transabdominal echocardiography in the Department of Ultrasound, The First Maternal and Infant Health Care Hospital, Shanghai, China, between March 2015 and December 2015. All fetuses were with normal cardiac functions (the stroke volume (SV): 0.23~1.21 ml, the ejection fraction (EF): 0.38~0.85). All echocardiographic examinations were performed with a GE VOLUSON 730 system (General Electric Healthcare, Milwaukee, WI, USA) with a RAB6-1 probe using the spatiotemporal image correlation (STIC) imaging mode. Each echocardiographic sequence spanned a cardiac cycle and contained 40 views. For the following analysis and comparisons, 3DE data was stored for each view, in which 2DE in 4-chamber view was extracted from the 3DE by an experienced doctor. The LV border for each 2DE four-chamber view was also delineated by the experienced doctor.

The study protocol was approved by the local ethics committees, and informed consent was obtained from all patients.

### 2.2. LV Volume References

**Figure 3:**
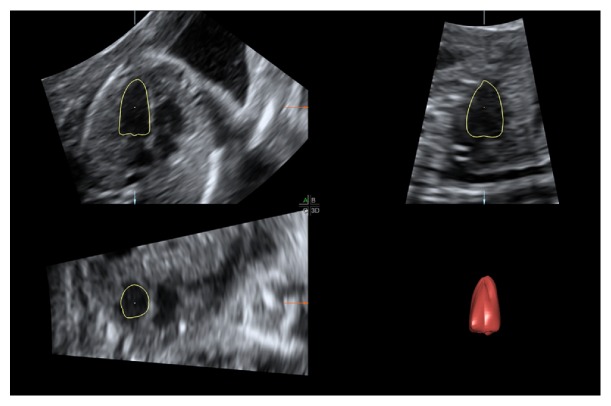
LV volume reference calculated by the VOCAL II mode in 4D view.

In this study, for LV volume references, we use the VOCAL II mode in 4D view version 14.0 (General Electric Healthcare, Milwaukee, WI, USA) as the volume calculation platform. As shown in Figure 3, it combines six 2D LV borders from different angles to rebuild the 3D LV and then calculate its volume [16, 17]. With enough angles, the volume computed by 4D view software is very close to the actual volume. It is always regarded as the references clinically.

### 2.3. BP Neural Network

Determination of fetal LV volume based on 2DE LV border is considered as a prediction problem which maps the nonlinear relations between the 2DE LV borders and the 3D LV volume. BP neural network which has strong nonlinear mapping ability is the most widely used method for prediction problem, so it can be used for the LV volume determination based on 2D LV border. Compared with the classical LV volume prediction methods which consider the LV as regular geometry model, BP neural network can construct more complex model which is closer to the real LV geometry model. So the BP neural network can predict the LV volume more accurately [18].

**Figure 4:**
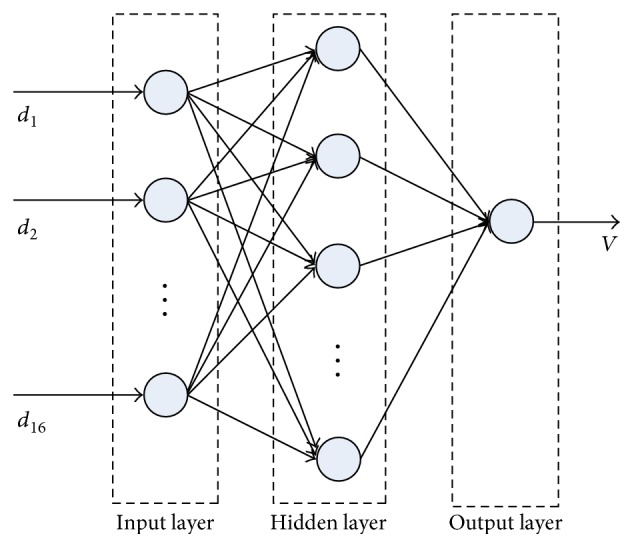
The architecture of a classic BP neural network.

The BP neural network is a multilayer feedforward one and trained by the error backpropagation algorithm. It is able to learn and store a lot of input-output mapping relations. There is no need to disclose in advance the mathematical equation that describes these mapping relations. As shown in Figure 4(), a classical architecture of BP neural network is composed of three layers: input layer, hidden layer, and output layer. The function of each node in BP neural network can be expressed as

(1) $$\begin{array}{c} o_{j,k}^{l}=f\left(\sum_{j}w_{j,k}^{l}o_{j,k}^{l-1}\right), \end{array}$$

where *l* represents number of layers, *o*_*j*,*k*_^*l*^ is the node in layer *l*, and *w*_*j*,*k*_^*l*^ is the corresponding weight. *f*(^∗^) is the transfer function. The most used transfer functions of BP neural network are the sigmoid function and pure-line function. The sigmoid function enables the BP neural network to approximate nonlinear mapping.

For determination of LV volume based on 2D LV border, the 2D LV border and the expected LV volume are considered as the input and output of BP neural network, respectively. The BP neural network is needed to be trained before calculating the LV volume directly, which searches for the optimal training parameters to improve the prediction precision. Usually, the BP neural network needs a large number of samples for training. In this study, 1000 samples are used for the training of BP neural network. The samples contain the input vectors and the expected output labels. In this study, the output labels are the volume references calculated from the VOCAL II mode by 4D view.

**Figure 5:**
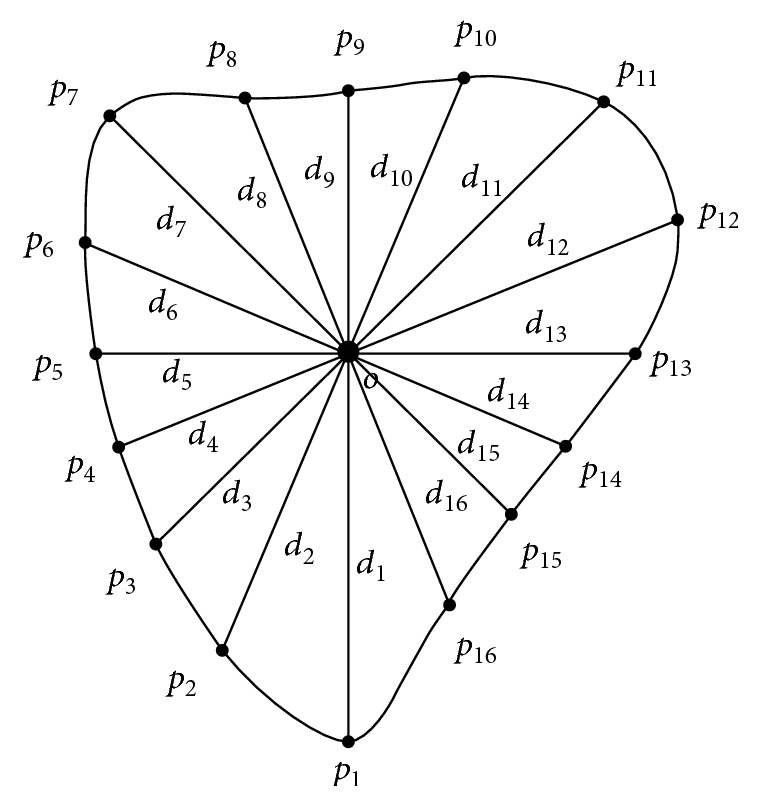
Distances for the BP neural network input. *O* is the center of LV. *p_i_* (*i* = 1, 2,…, 16) are the selected points on the border of LV with the equal angle. *d_i_* (*i* = 1, 2,…, 16) are the corresponding distances between *p_i_* and *O*.

In BP neural network, the input size of all samples must be the same. However, the size of the LV changes in different fetal echocardiographic sequences. If the entire 2D LV border is set as the input of the BP neural network, the input size is not uniform and the complexity will increase greatly. To unify and simplify the network, 16 distances are used as input of the network for each 2DE border. As shown in Figure 5, 16 distance values are calculated from 16 points on the border to the center of border. The 16 points on the border are selected by the equal angle, and the furthest point of the apes is the first point. Because the fetal LV is small and the border of LV is smooth, enough points can be used to represent the border. The 16 distances are starting from the same point and are selected by equal angle, so it is feasible to replace the border of LV with these distances.

**Figure 6:**
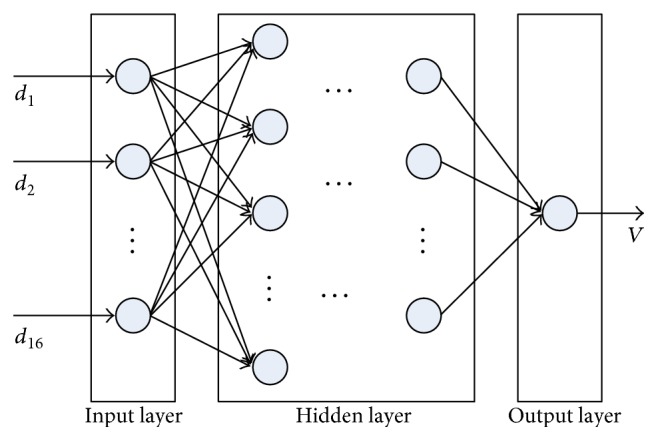
The structure of the BP neural network. *d_i_* (*i* = 1, 2,…, 16) are the distances, and *V* is the volume of LV.

With enough training samples, a BP neural network can be trained. A BP neural network with multiple hidden layers is proposed for fetal LV volume prediction. The structure of the BP neural network is shown in Figure 6. For hidden layers, the sigmoid function is used to obtain the nonlinear mapping. And for the output layer, the pure-line function is used to predict continuous LV volume. The performances of different number of hidden layers will be discussed in the following. The training process is composed of two parts: the information forward propagation and the error backpropagation, which aims to adjust the weights to make the error function of BP neural network achieve the minimum [19]. Here, half of views selected randomly were used for training the BP neural network. To evaluate the performance of the proposed method, the others were used for testing by the trained BP neural network.

### 2.4. Cardiac Function

The purpose of determining the fetal LV volume is to calculate LV function parameters, further to analyze the cardiac function. Based on the accurate LV volume prediction, two LV function parameters, the stroke volume (SV) and the ejection fraction (EF), can be computed. SV is determined as

(2) $$\begin{array}{c} SV=end‐diastolic\;volume-end‐systolic\;volume. \end{array}$$

EF is defined as

(3) $$\begin{array}{c} EF=\frac{SV}{end‐diastolic\;volume}. \end{array}$$

### 2.5. Compared Methods

To evaluate the performance of our proposed method, we compared it with some related algorithms, including (1) the area-length method, (2) Simpson's method, and (3) the multivariate nonlinear regression equation method.

For the area-length method, the volume of LV is given by the following expression [9]:

(4) $$\begin{array}{c} V=\frac{3}{4}\pi \cdot \frac{L}{2}\cdot \frac{D_{1}}{2}\cdot \frac{D_{2}}{2}. \end{array}$$

For single plane 2DE, *D*_1_ and *D*_2_ are equal and *D* can be calculated from the formula

(5) $$\begin{array}{c} D=\frac{4}{\pi }\cdot \frac{A}{L}, \end{array}$$

where *A* is the area of the LV in the 4-chamber view. So the volume of LV can be expressed as

(6) $$\begin{array}{c} V=\frac{8}{3}\cdot \frac{A^{2}}{\pi L}. \end{array}$$

For Simpson's method, the LV volume can be expressed as [10–12]

(7) $$\begin{array}{c} V=\frac{\pi }{4}\overset{h}{\underset{i=1}{\sum }}d_{1i}d_{2i}, \end{array}$$

where *h* is equal to the number of individual cylinders and *d*_1*i*_ and *d*_2*i*_ are equivalent to *D*_1_ and *D*_2_ of the ellipse formula. For single plane Simpson's method, *d*_1*i*_ and *d*_2*i*_ are equal and the volume is given by the formula as shown in Figure 2:

(8) $$\begin{array}{c} V=\frac{\pi }{4}\overset{h}{\underset{i=1}{\sum }}d_{1i}^{2}. \end{array}$$

For the multivariate nonlinear regression equation method, a quadratic regression equation is used. The inputs are the same as the BP neural network, and the equation is expressed as

(9) $$\begin{array}{c} V=c+\overset{16}{\underset{i=1}{\sum }}\left(a_{i}\cdot d_{i}^{2}+b_{i}\cdot d_{i}\right), \end{array}$$

where *d_i_*, *i* = 1, 2,…, 16, are the corresponding distances of 16 points to the center of border, *a_i_*, *i* = 1, 2,…, 16, are the corresponding quadratic term coefficients, *b_i_*, *i* = 1, 2,…, 16, are the corresponding monomial coefficients, and *c* is the constant coefficient.

Matlab R2011b (MathWorks, Natick, BSN, USA) was programed for all methods.

### 2.6. Statistical Analysis

In order to illustrate the performance of all methods, quantitative evaluations were calculated between the volume references and the result of each method. The bias and precision (limits of agreement) of four methods were compared by using Bland-Altman plot analysis on a pairwise basis with volume references. In Bland-Altman plot, each sample is represented on the graph by assigning the mean of the pairwise volumes as the abscissa (*x*-axis) value and the difference between the two volumes as the ordinate (*y*-axis) value. The intraclass correlation coefficient (ICC, 95% confidence interval (CI)) and the concordance correlation coefficient (CCC, 95% confidence interval (CI)) are two of the most popular measures of agreement for variables measured on a continuous scale. So, ICC and CCC were performed to evaluate the performance of each method. The ICC or CCC closer to 1 indicates a better LV volume prediction performance. MedCalc version 16.8 (MedCalc Software bvba, Romidepsin CAS Ostend, Belgium) was used for the calculation of ICC, CCC, and Bland-Altman plot. OriginPro 8 SR0 version 8.0724 (OriginLab Corporation, Northampton, MA, USA) was used to draw the line diagram for analysis.

## 3. Results

### 3.1. BP Neural Network Structure

For the BP neural network, the performances with different network structures are different from each other. Generally, the BP neural network with a simple network structure may lead to underfitting which will decrease the performance for both training and testing. However, the BP neural network with a complex network structure may lead to overfitting problems which will decrease the performance of testing. To obtain an optimal network structure, we compared the performances of 8 BP neural network structures with hidden layers from 1 to 8.

**Figure 7:**
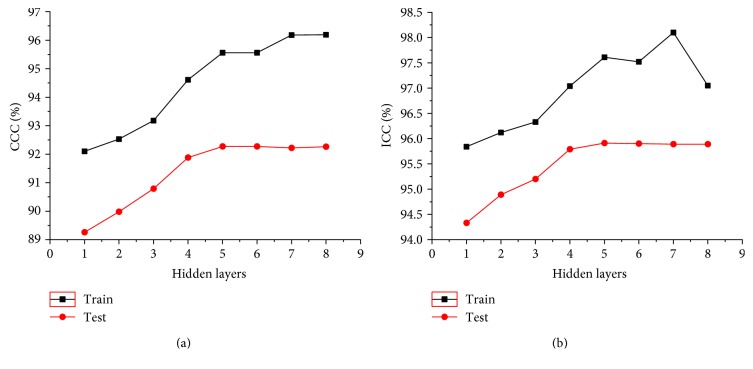
CCC and ICC between volume references and BP neural networks with different hidden layers.

Figure 7 shows the pairwise ICC and CCC at different hidden layers. In Figure 7(a), for the training data, CCC increased gradually from 1 to 8 hidden layers. However, for testing data, CCC varied slowly when the number of hidden layers reaches 4. In Figure 7(b), ICC of the training data also increased from 1 to 7 hidden layers, but it decreased rapidly at 8 hidden layers. While for testing data, their ICC varied slowly when the number of hidden layers reaches 4. So, with the increasing number of hidden layers, the performance for training increased gradually. But the inflection point appeared for testing performance when the number of hidden layers is 4. We also computed the operation time at different hidden layers, as shown in Table 1. Obviously, with the increase layers in the network structures, the operation time in training increased from 6.83 min to 21.43 min. Considering that the input of BP neural network is 16 distances, the time varied slowly for testing. Hence, we set the optimal number of hidden layers to 4. The structure of the BP neural network is shown in Figure 6, in which the number of hidden layers was set as 4.

### 3.2. Comparisons with Other Methods

**Figure 8:**
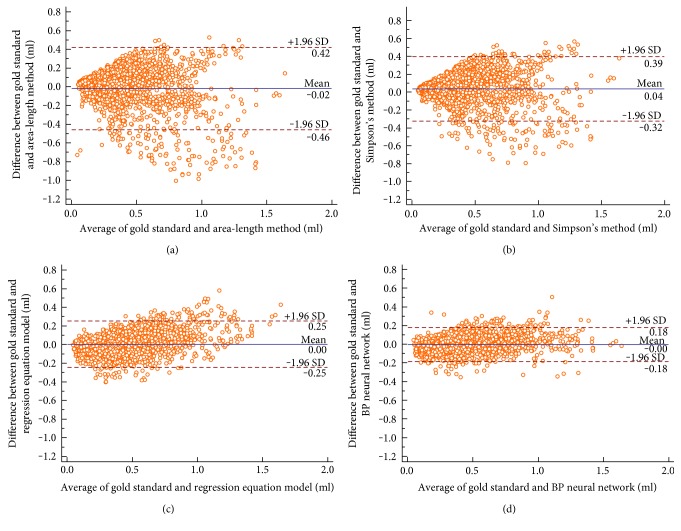
Bland-Altman plots of agreement between (a) the volume references and the area-length method, (b) the volume references and Simpson's method, (c) the volume references and the multivariate nonlinear regression equation method, and (d) the volume references and the BP neural network in volumes.

Figure 8 shows the pairwise Bland-Altman plots (95% CI for the bias) for volumes between each method and references. The average bias was 0 ml, and 95% CI was −0.18 to 0.18 ml between references and our BP neural network, while they were 0 ml and −0.25 to 0.25 ml for the multivariate nonlinear regression equation method, 0.04 ml and −0.32 to 0.39 ml for Simpson's method, −0.02 ml and −0.46 to 0.42 ml for the area-length method.

Table 2 shows the pairwise ICC (95% CI) and CCC (95% CI) by the different methods with volume references. In both ICC and CCC, the values for the BP neural network, the multivariate nonlinear regression equation, Simpson's method, and the area-length method decreased gradually. Similar trends can be also observed for 95% CI.

We also computed the average testing time for different method, as shown in Table 3. Due to the complex model, the average time for our BP neural network was 8.8 ms, which was longer than the other three methods. But, it is still short and clinically acceptable.

From Figure 8 and Table 2, the BP neural network outperforms other three methods with the best agreement. The area-length method shows the worst consistency.

### 3.3. Cardiac Function Analysis

**Figure 9:**
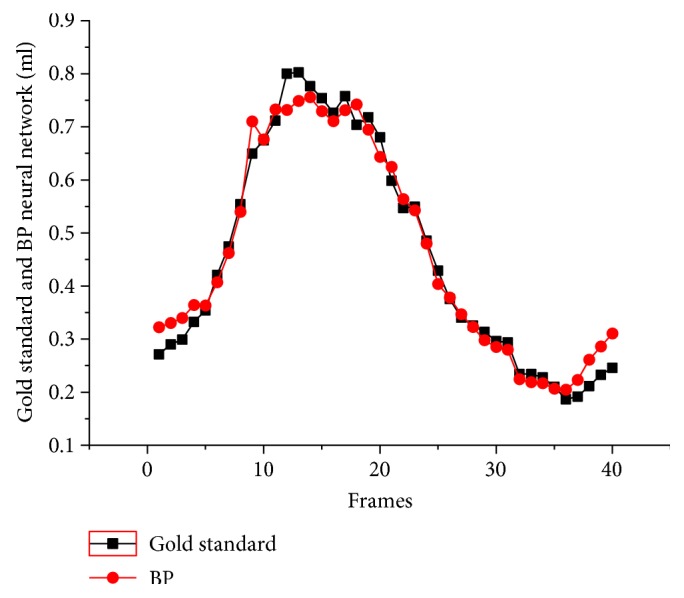
The variations of LV volumes for a case. The gold standard is the volume references.

With the movement of the fetal heart, its LV volume also varies at different cardiac cycle. As an example, Figure 9 is the LV volumes of successive views calculated by the BP neural network and 4D view software. It observed a high agreement between our method and the reference method. With the accurate LV volume calculated by the BP neural network, SV and EF can be calculated accurately.

**Figure 10:**
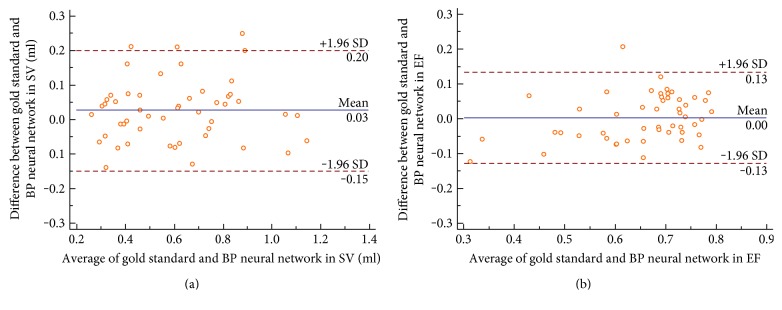
Bland-Altman plots of agreement between the volume references and the BP neural network (a) for SV and (b) for EF.

Figure 10 illustrates the pairwise Bland-Altman plots (95% CI for the bias) for SV and EF between our BP neural network and volume references. For SV, the average bias was 0.03 ml, and 95% CI was −0.15 to 0.20 ml. For EF, the average bias was 0 ml, and 95% CI was −0.13 to 0.13 ml.  Figure 10(b) demonstrates the consistence of EF between our method and the reference.

## 4. Discussion

### 4.1. Number of Hidden Layers in BP Neural Network

Robert Hecht and Nielsen proved that a continuous function at any closed interval can be approximated by a BP neural network with one hidden layer [14, 15]. However, considering the complicated LV volume prediction based on the 2DE LV border, the performance of one hidden layer BP neural network is limited. Generally, the increasing hidden layers will result in better prediction performance. However, the more complex network structure may lead to overfitting problems and higher operation time. A BP neural network with appropriate hidden layers is desired to balance these problems. Figure 7 and Table 1 illustrate that the BP neural network with 4 hidden layers achieved the balance between the agreement and reliability with volume references and complexity of network. When the number of hidden layers increases, both ICC and CCC increased for training data but varied slowly for testing data, which means an overfitting began to appear. Moreover, with the increasing number of hidden layers, the overfitting became more and more serious. So, it is reasonable that the number of hidden layers for the BP neural network was set to 4.

### 4.2. Compared with Other Methods

The area-length method calculates the LV volume based on the assumption that the LV is a completed ellipse. For Simpson's method, the LV is considered as the stack of individual short cylinders, which calculates from the furthest point of the apes to the mitral valve. However, the LV, which is a more complex geometry, is not a pure ellipse or the stack of individual short cylinders. Both the multivariate nonlinear regression equation method and BP neural network method consider that the LV is a complex geometry which is implicit in the regression equation or the neural network. So, as shown in Table 2 and Figure 8, the agreement and reliability with volume references for both the area-length method and the Simpson's method were smaller than those of the multivariate nonlinear regression equation method and BP neural network method.

Compared to a completed ellipse, the stack of individual short cylinders is more close to the LV geometry. Both ICC and CCC for Simpson's method with volume references were greater than those for the area-length method with volume references. As shown in Figures 8(a) and 8(b), the mean of difference and the 95% CI for Simpson's method with volume references were smaller than those for the area-length method with volume references. Therefore, the performance of Simpson's method is better than that of the area-length method.

The relative advantage of the multivariate nonlinear regression equation method and BP neural network method is the assumption that the LV geometry is implicit in the regression equation or the BP neural network. The multivariate nonlinear regression equation method represents the LV as a quadratic term regression equation. The BP neural network can represent more complex geometry than the multivariate nonlinear regression equation, which is closer to the real LV. As shown in Table 2, ICC and CCC between the BP neural network and volume references were greater than those for the multivariate nonlinear regression equation method with volume references. The advantage of BP neural network is also shown in Figures 8(c) and 8(d). In Bland-Altman plots, the 95% CI for BP neural network with volume references was smaller than that for the multivariate nonlinear regression equation method with volume references.

Therefore, the BP neural network shows the greatest performance for fetal LV volume determination based on single plane 2DE.

## 5. Conclusion

In this study, we proposed a novel method based on the BP neural network for the determination of LV volume in single plane 2DE. Different from conventional methods which consider the geometry of LV as ellipse or the stack of individual short cylinders, the geometry of LV is implicit in the BP neural network which can represent more complex geometry. Compared with other methods, the BP neural network have shown the best agreement and reliability with volume references. The CV and EF calculated from the BP neural network also showed the highest agreement with volume references. Therefore, for 2DE LV volume calculation, the BP neural network is reasonable and accurate.

## 6. Limitations

The limitation of this study is the small number of datasets. Small number of datasets may lead to underfitting, which will decrease the generalization ability of the BP neural network. More training data will be collected in the future to improve the robustness of our proposed method.

## Figures and Tables

**Table 1:** Operation time at different hidden layers of BP neural network.

Hidden layers	1	2	3	4	5	6	7	8
Training time (min)	6.83	8.64	10.51	13.72	16.93	17.38	19.68	21.43
Testing time (ms)	6.12	6.81	7.75	8.80	9.75	10.57	11.60	12.73

**Table 2:** The pairwise ICC (95% CI) and CCC (95% CI) of the different methods with volume references.

	ICC (95% CI)	CCC (95% CI)
Area-length versus volume references	0.8745 (0.8630~0.8851)	0.7756 (0.7603~0.7901)
Simpson's versus volume references	0.9025 (0.8936~0.9107)	0.8167 (0.8026~0.8299)
Regression versus volume references	0.9413 (0.9359~0.9413)	0.8794 (0.8695~0.8887)
BP versus volume references	0.9691 (0.9663~0.9717)	0.9401 (0.9348~0.9449)

**Table 3:** Average testing time for different methods.

Method	Area-length	Simpson's	Regression	BP
Testing time (ms)	0.81	1.58	2.02	8.8
